# Low pH alleviated salinity stress of ginger seedlings by enhancing photosynthesis, fluorescence, and mineral element contents

**DOI:** 10.7717/peerj.10832

**Published:** 2021-02-11

**Authors:** Fengman Yin, Shanying Zhang, Bili Cao, Kun Xu

**Affiliations:** 1 College of Horticulture Science and Engineering, Shandong Agricultural University, Tai’an, Shandong, China; 2 Collaborative Innovation Center of Fruit & Vegetable Quality and Efficient Production in Shandong, Tai’an, China; 3 Key Laboratory of Biology and Genetic Improvement of Horticultural Crops in Huanghuai Region, Ministry of Agriculture and Rural Affairs, P.R. China, Tai’an, China; 4 State Key Laboratory of Crop Biology, Tai’an, China; 5 College of Food Science, Hainan University, Haikou, Hainan, China

**Keywords:** Ginger, Salt stress, Low pH, Photosynthesis, Chlorophyll fluorescence, Mineral contents

## Abstract

We investigated the effects of low pH on the photosynthesis, chlorophyll fluorescence, and mineral contents of the leaves of ginger plants under salt stress. This experiment involved four treatments: T1 (pH 6, 0 salinity), T2 (pH 4, 0 salinity), T3 (pH 6, 100 mmol L^−1^ salinity) and T4 (pH 4, 100 mmol L^−1^ salinity). This study showed that photosynthesis (Pn, Gs, WUE and Tr) and chlorophyll fluorescence (qP, Φ PSII, and Fv/Fm) significantly decreased under salt stress; however, all the parameters of the ginger plants under the low-pH treatment and salt stress recovered. Moreover, low pH reduced the content of Na and enhanced the contents of K, Mg, Fe and Zn in the leaves of ginger plants under salt stress. Taken together, these results suggest that low pH improves photosynthesis efficiency and nutrient acquisition and reduces the absorption of Na, which could enhance the salt tolerance of ginger.

## Introduction

Soil salinity has severe effects on plant growth and development and plant productivity in arid and semiarid regions worldwide (Kochian et al., 2015; Krasensky & Jonak, 2012; Ryu & Cho, 2015). The increase in soil salinity and acidification is due to poor irrigation practices, improper fertilizer application, industrial pollution, and seawater intrusion caused by global warming (Gaudio et al., 2015; Ding et al., 2020). In particular, growers are advised to apply large amounts of chemical fertilizers for high yields during long-term vegetable production processes. This leads to a nutrient imbalance and excess salt accumulation in the soil. Salt causes several adverse effects on plant growth and development, including decreased leaf size, yellowing of the leaves, short internodes, short plant height, early flowering and decreased yields (Acosta et al., 2011; Thouvenot, Haury & Thiébaut, 2012; Jan et al., 2017; Ahmad et al., 2018)

Photosynthesis is an important biological process for maintaining plant life and plays a very important role in the evolution of ecosystems on Earth. Photosynthesis provides the energy and carbon required for the biosynthesis of organic compounds necessary for the growth and biomass production of plants. Increasing photosynthesis efficiency is critical to increasing crop yields to meet human demand for food (Long, Marshall-Colon & Zhu, 2015; Zhu, Long & Ort, 2010). Many researchers have studied the effects of salt stress on plant photosynthesis. There is considerable evidence for significant changes in the chlorophyll content (Chl) (Kalaji et al., 2016), net photosynthesis rate (Pn) (Jiang et al., 2017), stomatal conductance (Gs) (Janda et al., 2016), transpiration rate (Tr), maximum photochemical efficiency (Fv/Fm) (Singh, Singh & Prasad, 2016), amount of ribulose-1,5-bisphosphate carboxylase/oxygenase (RuBisco) and photochemical quenching (qP) (Moles et al., 2016). Chloroplast development and chlorophyll metabolism are important biological activities of photosynthesis in green plants. Studies have revealed chlorophyll synthesis-related enzymes and key regulators involved in chloroplast development (Bollivar, 2006; Cortleven & Schmulling, 2015). Tang et al. (2018) showed that NaCl stress significantly decreased the contents of Chl a, Chl b, and total chlorophyll in cucumber leaves. Ribose-1,5-bisphosphate ribulose carboxylation/oxygenase is a key enzyme that is involved in plant photosynthesis and controls both CO_2_ fixation and carbon. Rubisco is the key enzyme in the Calvin cycle, converting free CO_2_ in the atmosphere into energy-storage molecules, such as sucrose, and it plays a direct role in the photosynthesis rate.

Ginger (*Zingiber officinale* Rose), a perennial plant species of the family Zingiberaceae, is native to tropical rainforest regions. Ginger has been cultivated and widely used for more than 2000 years in China as a spice and as an important ingredient in traditional Chinese medicine. Because the bioactive constituents in ginger are valuable and have been accepted gradually by people (Ali et al., 2008; Li et al., 2015a), ginger’s demand has increased annually worldwide. According to statistics from the FAO (Food and Agriculture Organization of the United Nations), global ginger production was 813 340 tons in 2010 and 1 218 710 tons in 2016 but was 426 032 tons and 938 000 tons in mainland China, respectively.

Many studies on the photosynthesis and chlorophyll content of ginger have focused mainly on antibiotics and drought (Li et al., 2013; Li et al., 2015b; Liu et al., 2018). Salt stress was shown to decrease the growth and biomass yield (leaf fresh weight and root fresh weight) of ginger seedlings in a preliminary study. Moreover, low pH significantly alleviated this inhibition under salt stress, perhaps by lowering the Na content, alleviating osmotic stress, and enhancing plant nutrient uptake (Yin, Cao & Xu, 2019; Yin et al., 2020). However, several studies have focused on the effects of low pH on the photosynthesis and chlorophyll content of ginger under salt stress. As such, the objectives of this study were to investigate the changes in photosynthesis and chlorophyll content in response to acidic salt stress, which is important for understanding the mechanism underlying plant tolerance to acidic salt stress.

## Materials & Methods

### Plant materials and experimental treatments

The plant materials and experiment treatment reference Yin et al. (2020). In Tai’an, Shandong Province, China pot culture experiment was performed from April to October 2017. On May 14, the *Zingiber officinale* cultivar Shannong No. 1 were sown in pots (diameter, 25 cm; height, 30 cm) filled with cleaned quartz sand (Yin et al., 2020). Neutral salt stress was simulated with NaCl and Na_2_SO_4_(NaCl/Na_2_SO_4_=1/1), and pure water with different pH values (HCl/H_2_SO_4_=1/1) was used to simulate hydrochloric acid stress treatments. This experiment involved four treatments: T1 (pH 6, 0 salinity), T2 (pH 4, 0 salinity), T3 (pH 6, 100 mmol L^−1^ salinity) and T4 (pH 4, 100 mmol L^−1^ salinity). Each treatment was replicated three times, with six individual plants in each replicate. Each pot received 400 mL of treatment solution.

### Analytical methods

#### Photosynthesis parameters

Functional leaves of ginger were selected, and the Pn, Gs, Tr and Ci were measured by a portable photosynthesis system (Ciras-3, PP Systems, USA) using the method of Li et al. (2013), with slight modifications. When the Pn reached a steady state at each light intensity level, data were recorded 5 times per treatment, and the average value was calculated to determine the final photosynthesis parameters. Natural light was used, and the CO_2_ gas source was part of an open system.

#### Pigment concentrations

The chlorophyll content was measured according to the methods of Holm (1954).

#### Chlorophyll fluorescence

The qP, NPQ, ΦPSII, and Fv/Fm were measured according to the methods of Hendrickson et al. (2005) and Liu et al. (2018). At the time of measurements, 5 plants were averaged for each treatment.

#### Photosynthesis enzyme (RuBPCase, FBPase, and FBA) activity assays

Fructose 1,6-diphosphatase (FBPase) activity was measured according to the methods of Lazro et al. (1974).

RuBPCase activity was determined using an ELISA kit (Suzhou Keming), and fructose 1,6-bisphosphate aldolase (FBA) activity was determined using an ELISA kit (GenMed).

#### Sugar metabolism

Sucrose synthase (SS) and sucrose phosphate synthase (SPS) activity were estimated following the methods of Batta & Singh (1986).

Reducing sugars and sucrose contents were measured according to the methods of Handel (1968), using a standard graph of glucose.

The starch content was calculated according to the methods of Hannachi & Van Labeke (2018).

#### Mineral analysis

Ginger leaves were dried for 48 h at 75 °C and ground separately in a Wiley mill to pass through a 20-mesh screen. Afterward, 0.5 g of dried plant tissue was analyzed to determine the following major and minor elements: N, P, K, Ca and Mg. The nitrogen concentration in the plant tissues was determined by the Kjeldahl method after mineralization with sulfuric acid (Bremner, 1965).

Phosphorus concentrations were determined by titration with molybdenum antimony reagent in the presence of dinitrophenol (Shankar, Kumar & Agrawal, 2016).

K, Ca, Fe, Zn and Mg concentrations were determined by dry ashing at 400 °C for 24 h, dissolving the ash in 1/20 HNO_3_, and assaying the solution obtained using an inductively coupled plasma emission spectrometer (iCAP 7000 Series, Thermo Scientific).

#### Observations of ginger leaf chloroplast ultrastructure

Functional leaves were sampled (1 mm ×1 mm), quickly placed in a 2.5% glutaraldehyde fixative solution, and then transferred to a 4 °C refrigerator. The material was rinsed with 0.1 M PB (pH 7.4). Tissues avoid light post fixed with 1% OsO4 in 0.1 M PB (pH 7.4) for 7 h at room temperature., after which it was rinsed 3 times with 0.1 M PB (pH 7.4) again for 15 min each time. After that, the leaf tissue was then subjected to dehydration and infiltration, after which it was embedded. Afterward, the material was sectioned (Leica UC7), stained, the cuprum grids are observed under transmission electron microscope (HT7700, Hitachi) and take images.

### Statistical analysis

The data are presented as means ± one standard deviation (SD) for three independent replicates. The data were processed with DPS software. All graphs were created using the program SigmaPlot 10.0.

## Results

### Photosynthesis parameters

**Figure 1:**
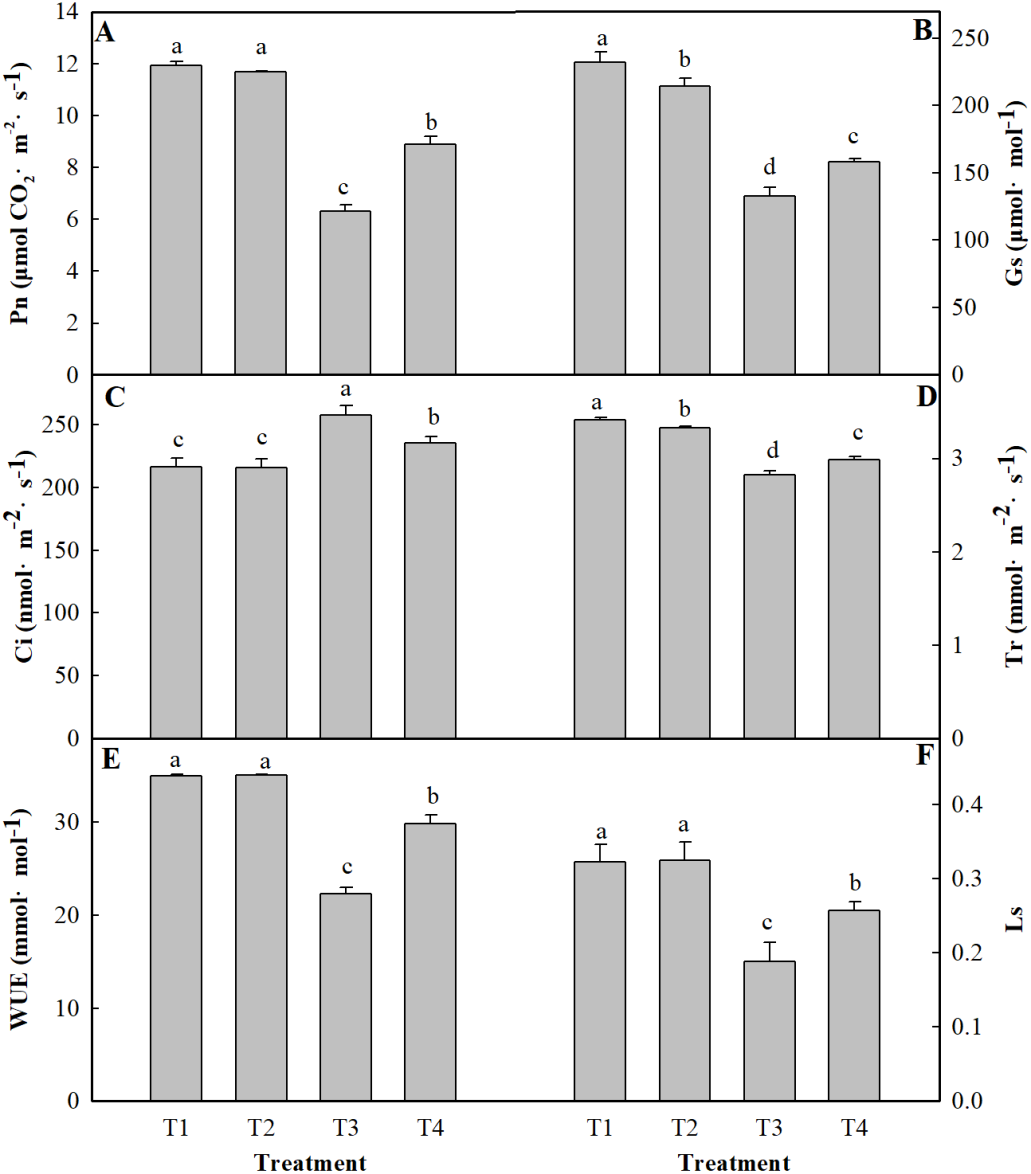
Effects of low pH on photosynthesis parameters of the leaves of ginger plants under salt stress. T1 (pH 6, 0 salinity), T2 (pH 4, 0 salinity), T3 (pH 6, 100 mmol L^−1^ salinity) and T4 (pH 4, 100 mmol L^−1^ salinity). The different small letters in a column of the same treatment days indicate significance at the 5% level.

Figure 1 shows the Pn, Gs, Ci, Tr, WUE and Ls of the leaves of ginger plants under salt stress with or without H^+^ application. Compared with those under the control treatment (T1), the Pn, Gs, Tr, and WUE decreased markedly (by 47.21%, 42.67%, 17.19%, 36.26%, respectively) under the T3 treatment, and the Ci increased by 18.92%. Moreover, compared with those under the control treatment (T1), the Pn, Gs, Tr, and WUE under the T4 treatment decreased by 25.42%, 31.90%, 12.50%, and 14.76%, respectively, and the Ci increased by 8.62%. Compared with that under the control treatment (T1), the Ls under the T3 and T4 treatments decreased by 41.75% and 20.26%, respectively. However, the pH treatment had only a certain effect on the Pn and WUE of ginger seedling leaves, but they did not significantly differ at the level of *P* < 0.05.

### Pigment contents

As shown in Table 1, compared with the control treatment (T1), the T3 treatment reduced the contents of Chl a, Chl b, Car, and Chl a+b by 9.33%, 28.28%, 7.59% and 13.01%, respectively. Moreover, the contents of Chl a, Chl b, Car, and Chl a+b slightly increased under the T4 treatment compared to the T3 treatment. There was no considerable difference caused by low pH in the Chl a, Chl b, Car, and Chl a+b contents in the leaves. Salt stress alone decreased the root activity by 26.94%. However, at low pH, salt stress decreased the root activity by only 19.57% (Table 2).

**Table 1:** Effects of low pH on Chlorophyll contents in ginger leaves under salt stress. T1 (pH 6, 0 salinity), T2 (pH 4, 0 salinity), T3 (pH 6, 100 mmol L^−1^ salinity) and T4 (pH 4, 100 mmol L^−1^ salinity). Different small letters in a column of the same treatment days indicate significance at the 5% level.

Treatment	Chl a (mg g^−1^ FW)	Chl b (mg g^−1^ FW)	Car (mg g^−1^ FW)	Chl a+b (mg g^−1^ FW)	Root activity (µg h^−1^ g FW)
T1	1.93 ± 0.01a	0.99 ± 0.0211a	0.79 ± 0.0122a	2.92 ± 0.013a	64.84 ± 1.36a
T2	1.8 ± 0.01b	0.82 ± 0.0049b	0.76 ± 0.0166b	2.62 ± 0.016b	61.29 ± 1.21b
T3	1.75 ± 0.01c	0.71 ± 0.0142c	0.71 ± 0.0068c	2.45 ± 0.017c	47.37 ± 0.62d
T4	1.78 ± 0.01c	0.74 ± 0.0102c	0.73 ± 0.0115c	2.51 ± 0.019c	52.15 ± 1.46c

**Table 2:** Effects of low pH on the activities of Rubisco, FBA and FBPase in ginger leaves under salt stress. T1 (pH 6, 0 salinity), T2 (pH 4, 0 salinity), T3 (pH 6, 100 mmol L^−1^ salinity) and T4 (pH 4, 100 mmol L^−1^ salinity) Different small letters in a column of the same treatment days indicate significance at the 5% level.

Treatment	Rubisco	FBA	FBPase
	nmol min^−1^ g^−1^	nmol min^−1^ g^−1^	nmol min^−1^ g^−1^
T1	105.65 ± 3.43a	155.81 ± 13.38a	81.23 ± 3.46b
T2	94.5 ± 2.28b	162.17 ± 6.07a	91.22 ± 4.51a
T3	65.35 ± 4.66d	116.65 ± 4.33c	65.55 ± 1.35c
T4	74.65 ± 0.83c	134.55 ± 8.67b	76.4 ± 3.26b

### Chlorophyll fluorescence

**Figure 2:**
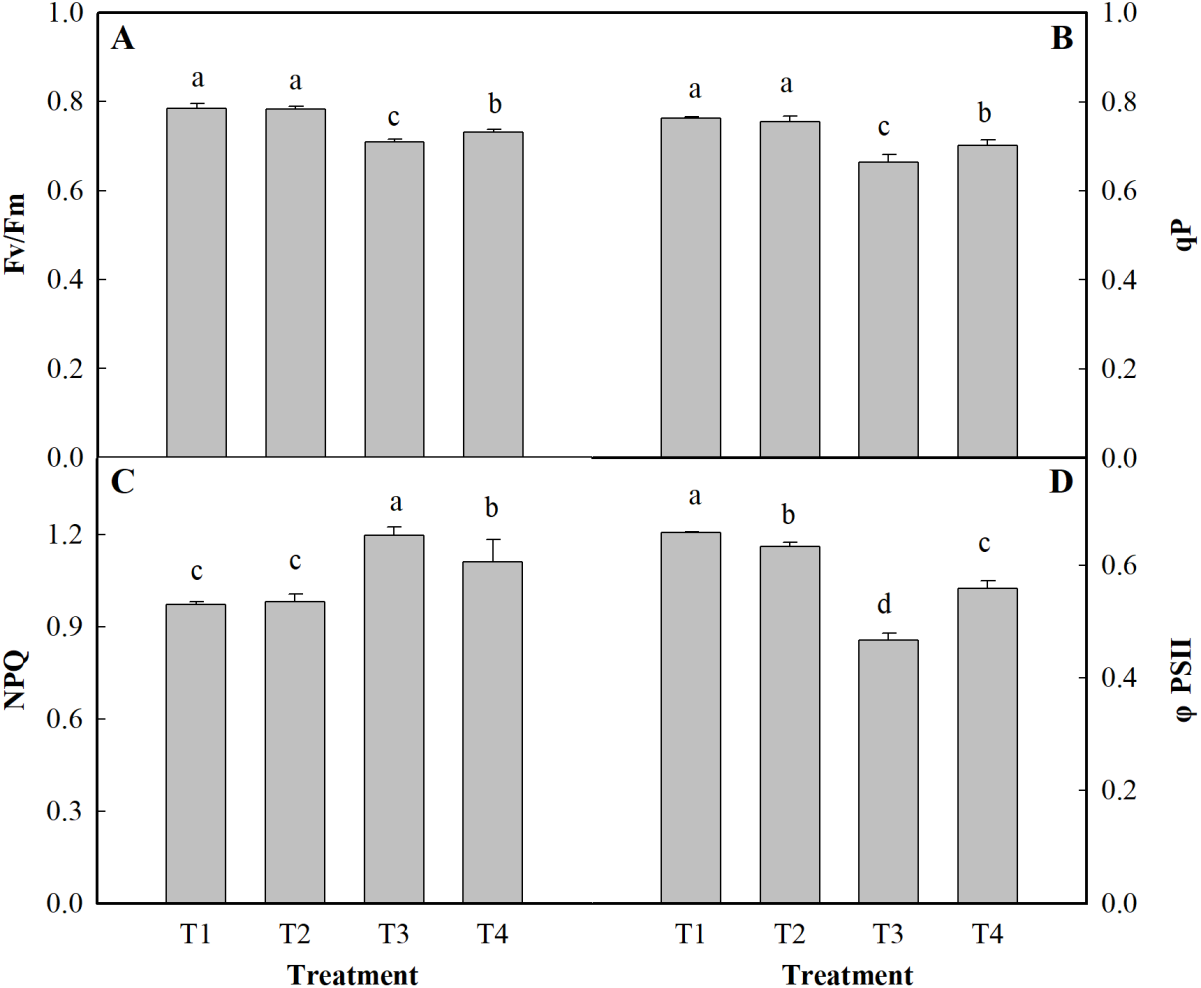
Effects of low pH on chlorophyll parameters of the leaves of ginger plants under salt stress. T1 (pH 6, 0 salinity), T2 (pH 4, 0 salinity), T3 (pH 6, 100 mmol L^−1^ salinity) and T4 (pH 4, 100 mmol L^−1^ salinity). The different lowercase letters in a column of the same treatment days indicate significance at the 5% level.

To analyze the changes in different ginger light systems in response to salt stress, chlorophyll fluorescence parameters were measured. These chlorophyll fluorescence parameters display significant negative effects under salt stress (Fig. 2). These effects were manifested by decreased Fv/Fm, qP, and ΦPSII values and an increased NPQ compared to the those of controls (the T3 and T4 treatments). Salt stress alone (T3) reduced the Fv/Fm, qP, and ΦPSII by 9.62%, 12.90%, and 28.96%, respectively, under the T3 treatment and by 6.85%, 7.87%, and 14.35%, respectively, under the T4 treatment compared to those under the normal conditions (T1). In contrast, the value of NPQ increased by 23.27% under the T3 treatment and by 14.35% under the T4 treatment compared to those under the control (T1).

### Photosynthesis enzyme activities

The results related to the activities of photosynthesis enzymes are depicted in Table 2. Low pH increased FBA and FBP activities by 4.08 and 12.30% and decreased Rubisco activity by 10.55% in the absence of salt stress, respectively, compared to those of the control seedlings (Table 2). Salt stress significantly decreased Rubisco, FBA, and FBP activities. Compared with the T3 treatment, the T4 treatment increased the Rubisco, FBA, and FBP activities by 14.23%, 15.34%, and 16.55%, respectively.

### Reducing sugar, sucrose, and starch contents

In the salt-affected ginger leaves, the reducing sugar, sucrose and starch contents decreased significantly (Table 3). Salt stress alone (T3) reduced the reducing sugar, sucrose, and starch contents by 51.75, 63.42, and 54.33%, respectively, compared to those of the control. However, at low pH, salt stress decreased reducing sugar, sucrose, and starch contents by only 35.06, 48.62, and 31.48%, respectively, compared to those under the T1 treatment. Table 3 shows that low pH had a weak effect on reducing sugar, sucrose, and starch contents, but they did not significantly differ at the level of *P* < 0.05.

**Table 3:** Effects of low pH on Reducing sugar, Sucrose and Starch content in ginger leaves under salt stress. DW stands for dry weight. T1 (pH 6, 0 salinity), T2 (pH 4, 0 salinity), T3 (pH 6, 100 mmol L^−1^ salinity) and T4 (pH 4, 100 mmol L^−1^ salinity) Different small letters in a column of the same treatment days indicate significance at the 5% level.

Treatment	Reducing sugar	Sucrose	Starch
	mg g^−1^ DW	mg g^−1^ DW	mg g^−1^ DW
T1	46.32 ± 0.73a	14.46 ± 0.59a	323.89 ± 2.68a
T2	40.51 ± 1.15b	12.31 ± 0.35b	319.44 ± 2.71a
T3	22.35 ± 1.05d	5.29 ± 0.08d	147.92 ± 5.12c
T4	30.08 ± 0.64c	7.43 ± 0.29c	221.94 ± 7.87b

### Activity of SS and SPS

**Figure 3:**
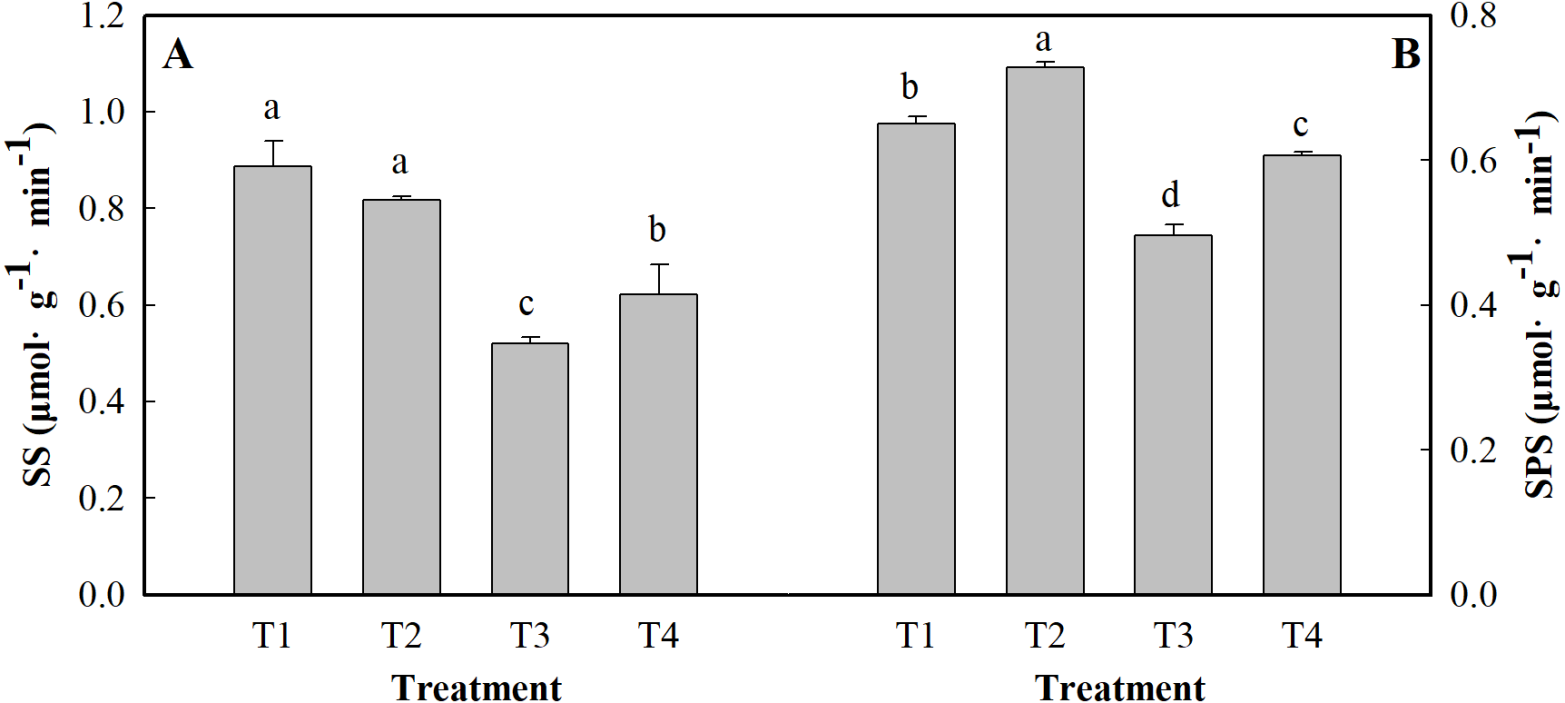
Effects of low pH on the activity of SS (A) and SPS (B) in the leaves of ginger plants under salt stress. T1 (pH 6, 0 salinity), T2 (pH 4, 0 salinity), T3 (pH 6, 100 mmol L^−1^ salinity) and T4 (pH 4, 100 mmol L^−1^ salinity). The different small letters in a column of the same treatment days indicate significance at the 5% level.

SS and SPS are key enzymes involved in carbon metabolism. The different treatments had significant effects on the activity of SS and SPS in the ginger leaves (Fig. 3). Salt stress alone reduced SS and SPS activity by 41.57 and 30.34%, respectively, compared to that of the control (T1). The activity of SS and SPS was reduced by 30.3 and 6.15%, respectively, under the salt +low pH treatment (T4) compared to the control treatment (T1).

### Content of phosphorus and nitrogen

**Figure 4:**
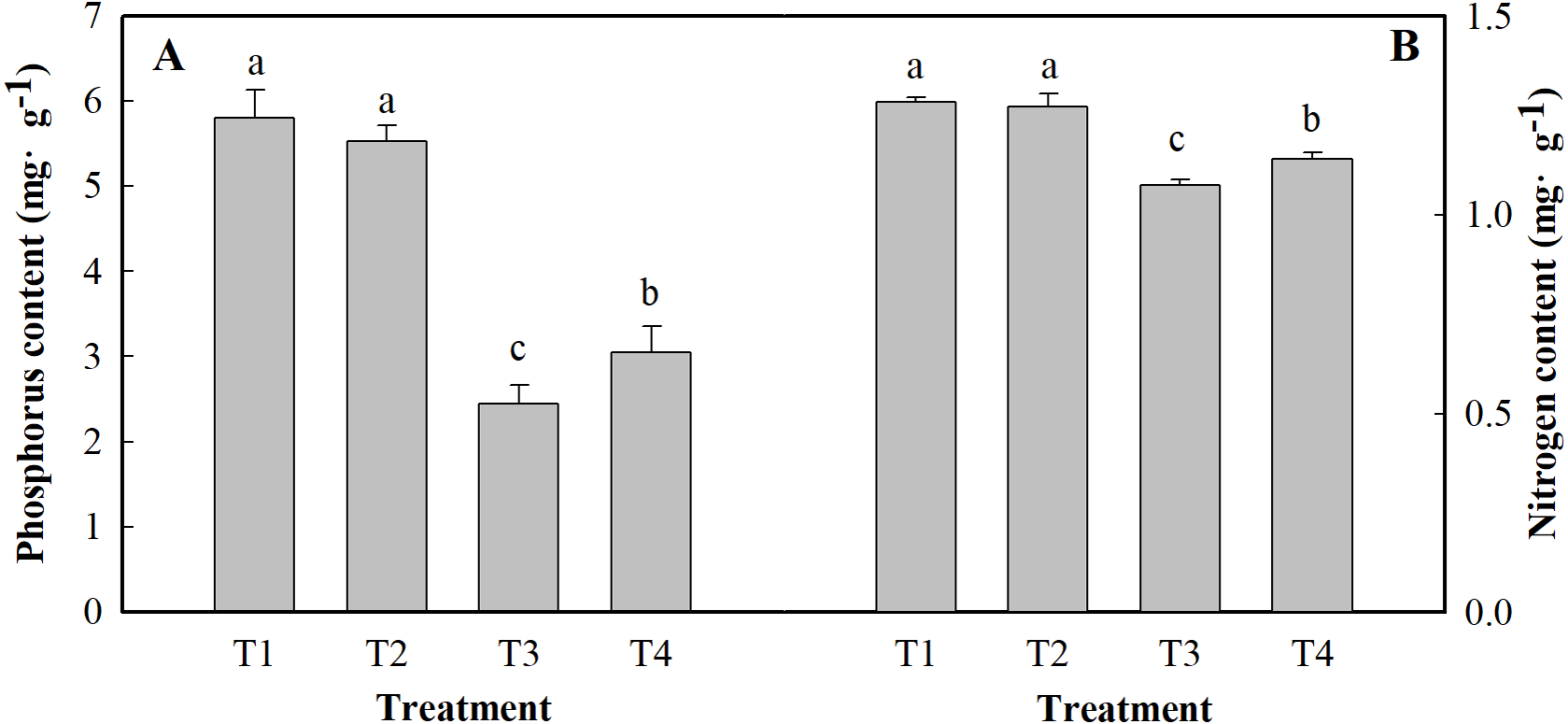
Effects of low pH on the contents of P (A) and N (B) in the leaves of ginger plants under salt stress. T1 (pH 6, 0 salinity), T2 (pH 4, 0 salinity), T3 (pH 6, 100 mmol L^−1^ salinity) and T4 (pH 4, 100 mmol L^−1^ salinity).

Figure 4 shows the results of the content of P and N in the leaves of ginger plants under salt stress or under low pH. Compared with the control (T1), salt stress alone (T3) reduced the content of P and N by 57.90 and 16.35%, respectively. Low pH with salt stress (T4) decreased the contents of P and N by 47.48 and 11.08%, respectively, compared to those of the control. Under low pH, the contents of P and N were slightly affected, but they did not significantly differ at the level of *P* < 0.05.

### Mineral composition content

**Figure 5:**
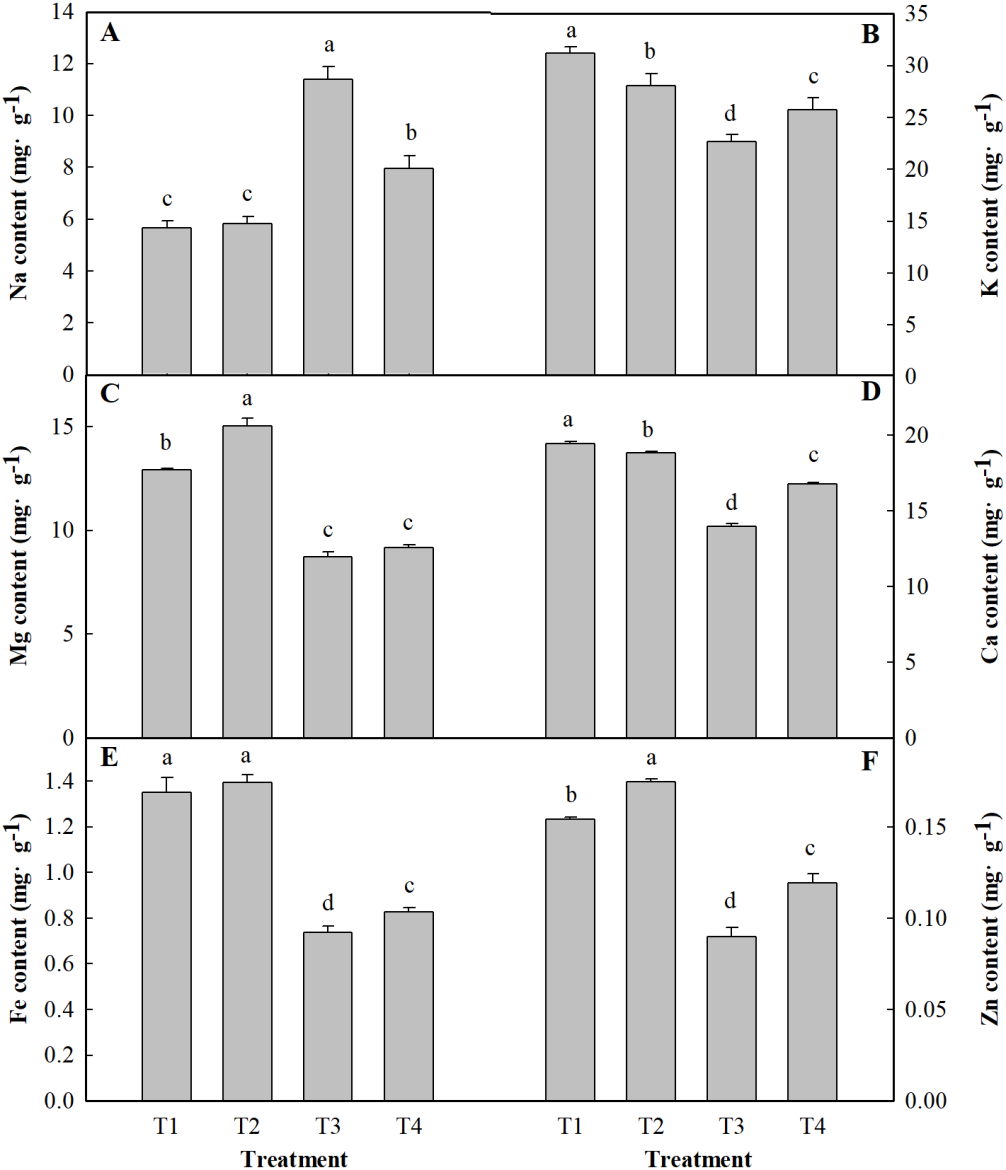
Effects of low pH on the contents of Na (A), K (B), Mg (C), Ca (D), Fe (E) and Zn (F) in the leaves of ginger plants under salt stress. T1 (pH 6, 0 salinity), T2 (pH 4, 0 salinity), T3 (pH 6, 100 mmol L^−1^ salinity) and T4 (pH 4, 100 mmol L^−1^ salinity).

As shown in Fig. 5, salt stress significantly increased the Na content in leaves of the plants and significantly decreased the contents of K, Mg, Ca, Fe and Zn. The content of Na in ginger seedling leaves increased by 101.55% under salt stress alone and increased by 40.62% under low pH and salt stress. Salt stress alone significantly reduced the contents of K, Mg, Ca, Fe and Zn by 27.27%, 32.52%, 28.08%, 47.01% and 41.73%, respectively, compared to those of the control. The contents of K, Mg, Ca, Fe and Zn decreased by 17.36, 29.38, 13.78, 40.64 and 31.75%, respectively, under low pH with salt stress (T4), respectively, compared to those under the control treatment (T1).

### Ultrastructure morphological changes

**Figure 6:**
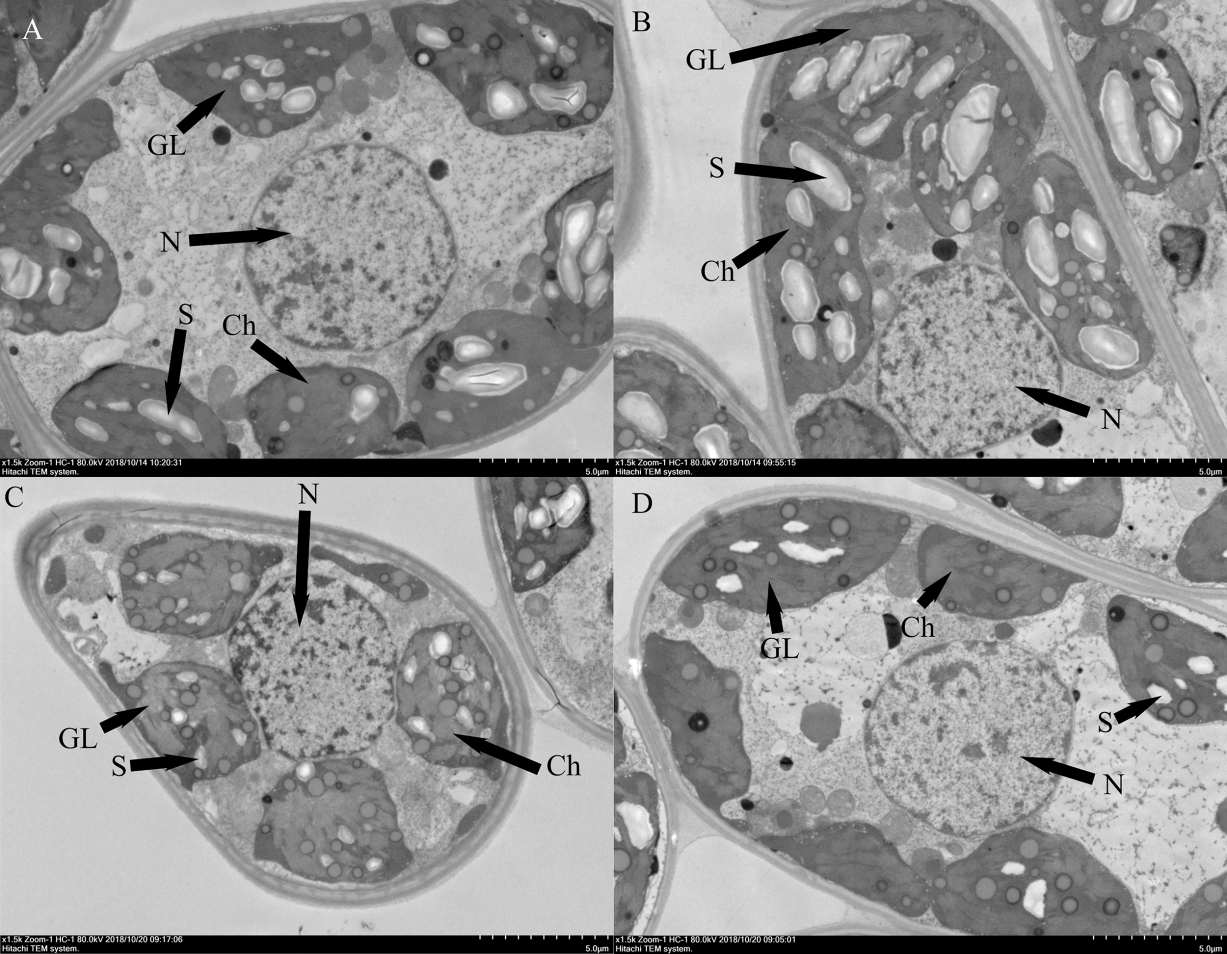
Effects of low pH on the leaf ultrastructure in the leaves of ginger plants under salt stress: T1 (pH 6, 0 salinity), T2 (pH 4, 0 salinity), T3 (pH 6, 100 mmol L^−1^ salinity) and T4 (pH 4, 100 mmol L^−1^ salinity). GL: Granum lamellae; S: Starch grains; ch: Chloroplast; N: Cell nucleus.

Changes in whole mesophyll cells and chloroplasts are shown in Fig. 6. Seedlings grown under normal conditions exhibited regular cell shape and typical chloroplasts; moreover, there were several well-packed starch grains (Fig. 6). However, cell morphological disturbance and plasmolysis occurred when the seedlings were treated with salt stress alone. The shapes of chloroplasts were severely swollen. Furthermore, there was an abundance of osmiophilic granules and fewer starch grains in the chloroplasts compared with those of control (Fig. 6). For low-pH-treated seedlings under salt stress conditions, there was a small improvement in cell morphology. The chloroplasts contained more starch grains than did those of seedlings under salt stress alone (Fig. 6).

## Discussion

### Photosynthesis

The damage caused by salt to plants is primarily attributed to the inhibition and disruption of photosynthesis, and the decrease of photosynthetic efficiency is one of the important reasons for the decrease of plant biomass under salt stress. The Pn decreases because of stomatal limitation or nonstomatal limitation. If Gs and Ci are positively correlated, then the reason for the Pn decrease is related to stomatal limitation; if the two showed no correlation or contrast, then it is related to nonstomatal limitation. In the present experiment, the Pn and Gs decreased under the T3 treatment, and the Ci increased. This suggests that the main factor of photosynthesis limitation is nonstomatal limitation. This is consistent with the research results of Tang et al. (2018). However, damaged photosynthetic structures may be another factor affecting the photosynthesis rate. The photosynthesis enzyme (Rubisco, FBA, and FBP) activities were related to the degree of damage to the photosynthetic structure. 1,5-Ribulose diphosphate oxygenase/shuttle enzyme is an important enzyme involved in CO_2_ fixation in plant leaves and plays an important role in maintaining photosynthesis (Parry et al., 1997). In this study, the activity of Rubisco, FBA and FBP decreased under the T3 treatment. These results suggest that the reason for photosynthesis under salt stress alone is nonstomatal limitation. Similar results were reported previously (Feng et al., 2014; Ning et al., 2018) However, low pH with salt stress reduced the Ci and increased the Pn; Gs; and the activities of Rubisco, FBA and FBP. Taken together, these results indicated that low pH could protect photosynthetic structures and increase the photosynthesis enzyme activities, thereby increasing the photosynthesis rate.

The water-use efficiency (WUE) of leaves is an important factor affecting whether plants can adapt to extreme environmental conditions (Martin, Tauer & Lin, 1999). In our experiment, the WUE value decreased under salt stress alone. This decrease was related to the reduced leaf transpiration rate, which was caused by the decreased Gs (Bacha et al., 2017). These conditions were unfavorable for substance transport in ginger. However, low pH increased the WUE of leaves under salt stress and improved substance transport in ginger.

### Chlorophyll fluorescence

The absorption and transformation of light energy by plants are mainly divided into three closely related parts: chlorophyll fluorescence, qP-related photosynthetic electron transport, and qN-related heat consumption (Schreiber, Schliwa & Bilger, 1986). As an important physiological index for evaluating plant growth and development characteristics, the chlorophyll content can reflect plant health and adaptability of plants (Guo et al., 2015; Xiao, Sang & Wang, 2008). Salt stress causes the decrease of chlorophyll and carotenoid contents in mung bean leaves, which may be caused by the expansion of chloroplast membrane and/or excess Na^+^ ions in the leaves (Alharby et al., 2019). In this study, the levels of Chl a, Chl b, and carotenoids decreased under salt stress alone. This is consistent with the research results of Do et al. (2018) and Patil et al. (2016). Chlorophyll content has an obvious correlation with the photosynthesis ability of leaves (Dhanapal et al., 2016), and a decrease in chlorophyll content can lead to an irreversibly decreased photosynthesis rate.

The maximal photochemical efficiency of PSII (Fv/Fm) was used to evaluate the primary conversion efficiency of light energy in the PSII reaction center (Jagerbrand & Kudo, 2016). ΦPSII is the actual light-harvesting efficiency of PSII when the reaction center is partially closed, and ΦPSII reflects the ratio of energy consumed by photosynthetic transmission of electrons when the leaves absorb energy. The qP reflects the relative proportion of light energy captured by light-harvesting pigments for photochemical electron transfer (Awlia et al., 2017). The values of Fv/Fm, ΦPSII, and qP were significantly reduced under salt stress, suggesting that salinity induced the inhibition of PSII electron transport and dissipated the excess excitation in the form of heat. This resulted in the reduction in the fluorescence quantum yield (Mehta et al., 2010). The decrease in qP also indicated an increase in the fraction of reduced QA in PSII (Bacha et al., 2017; Hu, Yan & Yu, 2016). The NPQ value, which represents heat dissipation, increased by 23.27%, which indicated that a greater share of excess energy was released as heat, whereas the ability to utilize light energy decreased. The Fv/Fm, ΦFPSII, and qP values increased significantly under low pH with salt stress, indicating that the photochemical activity and electron transfer in ginger leaves were positively affected and thereby enhanced the light energy conversion efficiency of PSII.

### Metal elements

Salt stress leads to specific ion toxicity and plant growth inhibition (Park, Kim & Yun, 2016). Excessive accumulation of Na^+^ is harmful to plant cells, which can lead to significant changes in metabolism and malnutrition (Liang et al., 2018). Nitrogen is an important major element in plant growth and development. It is a component of many plant cell components, including amino acids, proteins, and nucleic acids. The results show that salt treatment induced a decrease in N concentration in ginger plants in our study. However, the plants that were grown under low pH had consistently higher N concentration than the normal plants under salt stress. The decrease in N concentration due to salt stress may be caused by interference by salinity in N acquisition and utilization. Our study corroborates the findings of Zhang et al. (2016), who reported that phosphorus concentration decreased under salt stress. The effect is compounded by the deficiency of other elements (K, Mg, etc.) due to the excessive Na content, which also severely reduces photosynthesis (Lu et al., 2017). In this study, as expected, in the salt-stressed plants, Na accumulated excessively in the leaves. On the other hand, the K content under salt stress alone was markedly lower than that without salt stress treatment, which implied that there is a competitive relationship between K^+^ and Na^+^ in ginger leaves. A similar result was reported by Wakeel et al. (2011). However, low pH with salt stress decreased the Na content and increased the K content. Moreover, the results showed that low pH with salt stress resulted in a stronger ability for the absorption and transport of K^+^ to ensure an adequate concentration of the ions that participate in key metabolic activities (e.g., photosynthetic metabolites) in leaves. As is well known, Mg also plays an important role in photosynthetic metabolism. Mg accumulations were shown to be significantly positively correlated with the relative photosynthesis rate under salt stress (Ning et al., 2018). In the present study, the Mg content decreased under salt stress alone. These results suggest that salt stress reduced chlorophyll concentrations and photosynthesis by imparting a negative impact on Mg^2+^ uptake. Under salt stress, the concentrations of micronutrients (Fe and Zn in) ginger leaves decreased. Similarly, the concentration of Fe and Zn in chickpea plants decreased with NaCl stress, as reported by Shankar, Kumar & Agrawal (2016). However, compared with salt stress alone, low pH with salt stress resulted in a significantly higher concentration of micronutrients in plants. This may be attributed to the low pH reducing the Na content and thus enhancing the absorption of trace micronutrients.

**Figure 7:**
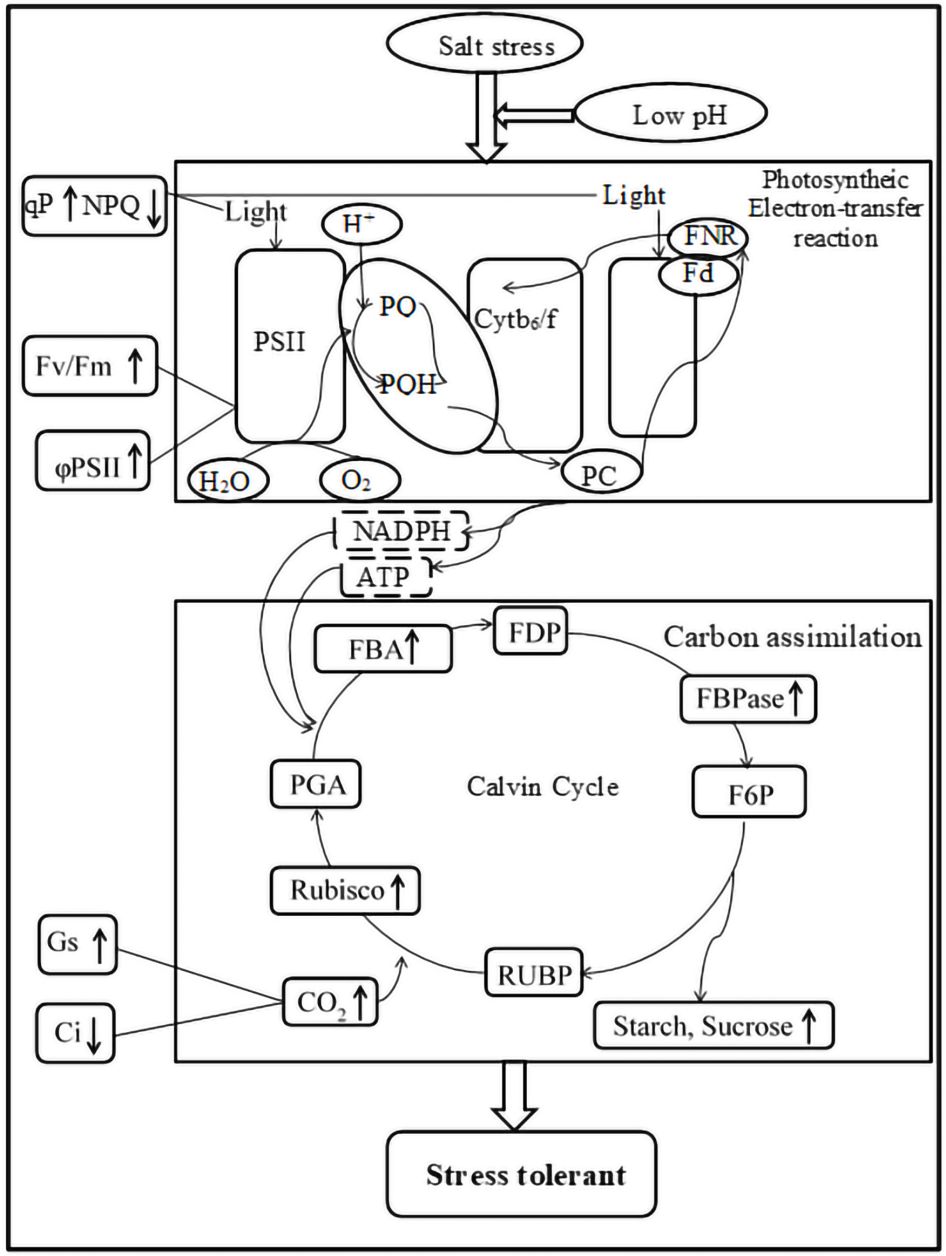
Impacts of low pH on photosynthesis processes of ginger under salt stress. “ ↓ ” indicates a decrease, “ ↑ ” indicates an increase.

## Conclusions

Salt stress is one of the major abiotic stresses that inhibit plant growth. As shown in Fig. 7, salt stress significantly inhibited the growth and decreased the photosynthesis, pigment contents and mineral contents of ginger leaves. Low pH with salt stress enhanced the activities of RuBPCase, FBPase, and FBA and increased the pigment contents, increasing the photosynthesis rate. Moreover, it is worth noting that low pH simultaneously increased the accumulation of K, Mg, Ca, Fe, and Zn. In summary, the improvement of photosynthesis, pigment contents, and accumulation of minerals due to low pH ultimately increased the biomass accumulation of ginger seedlings under salt stress.

##  Supplemental Information

10.7717/peerj.10832/supp-1 Supplemental Information 1 Raw measurements.

## Additional Information and Declarations

### Data Availability

The following information was supplied regarding data availability: Raw measurements are available as a Supplemental File.
